# Targeting CD83 for the treatment of graft-versus-host disease

**DOI:** 10.3892/etm.2013.1033

**Published:** 2013-04-02

**Authors:** XIONGFEI WANG, MING Q. WEI, XIAOSONG LIU

**Affiliations:** 1 Division of Molecular and Gene Therapies, Griffith Health Institute and School of Medical Science, Griffith University, Gold Coast, Queensland 4222, Australia;; 2 Cancer Research Institute, Foshan First People’s Hospital, Foshan, Guangdong 528000, P.R. China

**Keywords:** CD83, sCD83, antibodies, graft-versus-host disease

## Abstract

Graft-versus-host disease (GVHD) is a common and often fatal complication of bone marrow transplantation. Antigen-presenting cells from donor and recipient play a critical role in the initiation and maintenance of GVHD. CD83, which is expressed in activated lymphocytes and dendritic cells, is regarded as a marker of mature dendritic cells. Targeting CD83 using soluble CD83 molecules or antibodies has been demonstrated to have therapeutic effects against GVHD in preclinical models. Understanding the biological function of CD83 and the underlying mechanisms through which targeting CD83 attenuates GVHD is likely to greatly improve current treatments and provide new methods for the treatment of GVHD.

## Contents

1. Introduction
2. CD83 protein structure
3. Expression and functional roles of CD83 on lymphocytes and dendritic cells (DCs)
4. Soluble form of CD83 has an immune-regulatory function
5. Targeting CD83 using antibodies *in vivo* has therapeutic effects in different animal models
6. Targeting CD83 for the management of graft-versus-host disease (GVHD) and possible underlying mechanisms

## 1. Introduction

CD83 was first described in early 1990s (1) in Langerhans cells and activated lymphocytes. CD83 belongs to the immunoglobulin (Ig) superfamily and is a highly glycosylated type I transmembrane glycoprotein composed of 175 amino acids in the mouse and 186 in the human equivalent. Studies in CD83 knockout mice reveal that CD83 is essential for thymic maturation, peripheral function and longevity of CD4^+^ T cells (2); CD83 is also involved in B-cell maturation, peripheral B-cell function and homeostasis (2). Despite its expression on a number of cell types, CD83 is best known as a DC maturation marker (3). Other cells that express CD83 include activated macrophages, a subset of natural killer (NK) cells, activated neutrophils and thymic epithelial cells. In the peripheral immune system, CD83 inhibits the proliferation and production of interleukin (IL)-2 and interferon (IFN)-γ by T cells, which is mediated by monocytes (4).

Haematopoietic cell transplantation (HCT) is an effective therapy used to treat a range of haematological and genetic disorders. However, a common and often fatal complication of HCT is the induction of GVHD.

GVHD arises when donor T cells respond to genetically defined proteins on host cells, and is a key contributor to the high mortality associated with HCT. GVHD is summarised in three sequential steps or phases: i) activation of antigen-presenting cells (APCs); ii) donor T-cell activation, proliferation, differentiation and migration; and iii) target tissue destruction (5). The activation of donor and recipient APCs plays critical roles in the initiation of GVHD. Host APCs alone are sufficient to activate donor T cells in minor and major histocompatibility complex (MHC) mismatch settings (6). In full MHC or single MHC disparate murine bone marrow transplants, antigen presentation by host APCs also plays an important role in the induction of graft-versus-leukemia (GVL) (7,8). Host Langerhans cells are responsible for GVHD within the skin, as depletion of these cells prevents skin GVHD (9). Donor APCs take up antigens via the indirect pathway, predominantly via MHC class II to CD4 T cells. Donor conventional DCs are the primary APC responsible for indirect presentation of alloantigens following bone marrow transplantation (BMT) and this process commences almost immediately after transplantation (10). Recipient B cells do not appear to invoke alloantigen-dependent donor T cell responses and instead are likely to have a regulatory role, which is IL-10-dependent (10). Notably, donor NK T cells in HCT suppress GVHD induced by donor CD4 and CD8 conventional T cells (11). A previous study demonstrated that surfactant protein A (SP-A) protects against the development of gastrointestinal GVHD and establishes a role for SP-A in regulating the immune response in the gastrointestinal tract (12). It was identified in a murine model that GVHD prevents the maturation of plasmacytoid DCs (13), while a clinical study identified that myeloid and plasmacytoid DCs in the peripheral blood significantly upregulate CD83 on their cell surface following BMT (14), suggesting that a mouse model may not sufficiently represent clinical situations. Clinically, a study demonstrated that the C-C chemokine receptor 5 (CCR5) antagonist maraviroc inhibits CCR5 internalisation and lymphocyte chemotaxis *in vitro* without impairing T-cell function or the formation of haematopoietic cell colonies, suggesting that the inhibition of lymphocyte trafficking is a specific and potentially effective new strategy for preventing visceral acute GVHD (15).

Furthermore, although there are no reliable diagnostic tests to predict treatment, with the exception of six previously validated diagnostic biomarkers of GVHD (IL-2 receptor-*α*; tumour necrosis factor receptor-1; hepatocyte growth factor; IL-8; elafin, a skin-specific marker; and regenerating islet-derived 3-*α*, a gastrointestinal tract-specific marker) (16), a novel acute GVHD risk score has been created to define high-risk acute GVHD at the onset (17).

As a therapeutic target, CD83 has demonstrated utility in a variety of applications, from the use of soluble, recombinant CD83 in models of experimental autoimmune encephalomyelitis (EAE) and skin transplantation (18), to the use of depletion antibodies in the prevention of GVHD and giant-cell arteritis (GCA) in animal models. In this review, we summarise literature regarding the biological function of CD83 and potential clinical applications targeting CD83, with an emphasis on targeting GVHD. We further speculate on possible mechanisms targeting CD83 for the treatment of GVHD.

## 2. CD83 protein structure

The mature form of human CD83 is composed of 186 amino acids with residues 1-94 showing significant IgV-like homology. The molecule retains significant evolutionary homology, although certain parts of the protein are more conserved than others.

Although its crystal structure has not been determined, CD83, like other V-domain proteins, is considered to form an Ig loop via a disulphide bridge between Cys15 and Cys87. A third, highly conserved cysteine at position 109 forms an inter-molecular disulphide bond resulting in homo-dimerisation of recombinant human CD83 (19). Two additional cysteine residues within the human sequence at positions 7 and 80 have been postulated to form an intramolecular disulphide bond. However, these residues are not as strictly conserved; Cys80 is absent from the canine sequence, which possesses a serine at this position. This indicates that Cys7 and Cys80 in human CD83 do not interact to form a disulphide bond or that the disulphide bond is not structurally critical for activity.

N-linked glycosylation has also been identified as a key feature of membrane CD83 and is required for surface expression on DCs (20). Whilst glycosylation may affect surface expression, CD83 expressed in *Escherichia coli* does not appear to be structurally different from mammalian expression systems as determined by nuclear magnetic resonance (21). Furthermore, the functional activity of the prokaryotic product indicates that N-linked glycans are not essential in the mechanism of CD83 *in vitro*(19). These findings support similar observations for other Ig superfamily members, including CD2, where structural integrity and ligand binding functionality are glycosylation-independent (22).

The intracellular domain of CD83 possesses a greater degree of homology among species. For example, whilst full-length mouse and human CD83 share 60% amino acid homology, the intracellular domain is >80% homologous. A previous study (23) elucidated a degradation mechanism of CD83 through the presence of four conserved lysines, which are targets for ubiquitination. Once tagged with ubiquitin, integral membrane proteins are endocytosed and degraded in the lysosome (24,25). CD83 does not appear to undergo rapid recycling to and from the plasma membrane; thus ubiquitination provides an important downregulation mechanism.

In addition to downregulation, a soluble form of CD83 present in peripheral blood is considered to serve as an immunomodulator of the CD83-ligand interaction (26). It has been suggested that soluble CD83 (sCD83) may be derived from proteolytic cleavage of membrane-bound CD83 or from an alternate splice variant. To date, no evidence of the former possibility has been uncovered, other than the observation that sCD83 in the supernatant of KM-H2 cells possesses a slightly lower molecular weight (27).

Alternative splice variants of CD83, however, hold an interesting proposition. Of the three postulated CD83 splice variants, only variant C creates a translated protein (28). This protein contains only the first 50 amino acids of the extracellular domain and the intracellular portions of CD83. Tests of this variant (28) identified that, like sCD83, it is capable of a functional blockade of the CD83-ligand interaction; this implies that the ligand-binding domain of CD83 lies proximal to the N-terminus of the protein. However, this splice variant differs significantly from the full-length protein; not only is it approximately half the size of the full-length protein, but it does not contain the second cysteine traditionally used in IgV-loop formation. Often this loop is critical in ligand recognition for numerous Ig superfamily members, including killer cell inhibitory receptors (KIRs) (29), CD2 (30) and SHPS-1 (31). Further studies into CD83 and potential ligand(s) are required.

## 3. Expression and functional roles of CD83 on lymphocytes and dendritic cells (DCs)

### T cells

Resting CD4 T cells lack CD83 surface expression; although CD83 mRNA is detectable in naive CD4 T cells, particularly in CD4^+^CD25^+^ T cells. CD4^+^CD25^+^ T cells have almost 10-fold higher mRNA levels compared with CD4^+^CD25^−^ T cells (32). CD83 is upregulated on the majority of T cells within hours of activation *in vitro*(3). Previously, the degradation of CD83 by gene related to anergy in lymphocytes (GRAIL) via ubiquitination and subsequent proteolysis has been identified (23). CD83 is absent from CD8 T cells and is not upregulated following stimulation *in vitro*(32).

Early studies suggested that CD4^+^ T cells from CD83 knockout mice proliferate normally (33). However, numerous studies contradict this initial supposition. For example, analysis of CD83 knockout mice reveals that CD83 has a central function in the thymic selection of double-positive thymocytes to CD4 single-positive T cells, as demonstrated by a significant reduction in CD4^+^ cells in this mouse model. Also, CD4^+^ T cells in the CD83 knockout fail to respond normally following allogeneic stimulation (34) and similarly, knockdown of CD83 by RNA interference (RNAi) diminishes antigen-specific CD4 T-cell proliferation (23). Transduction of naive CD4^+^CD25^−^ T cells with CD83 encoding retroviruses induces a regulatory phenotype *in vitro,* with the upregulation and expression of Foxp3 (32). These cells have the capacity to increase anti-inflammatory cytokine IL-10, suppress pro-inflammatory cytokines, including IFN-*γ* and IL-17 and prevent the paralysis associated with EAE in recipient mice. A transgenic mouse expressing a soluble mCD83 IgG1 fusion protein under the control of the MHC class II promoter was generated (35). CD4^+^ T cells from mCD83IgG1/OTII (CD4 T cells specific for MHC II-restricted OVA peptide) double transgenic mice have reduced proliferation and IL-2 and IFN-*γ* production in response to wild type APCs, and mCD83 IgG1 transgenic mice infected with *Trypanosoma cruzi* exhibit a higher parasitaemia and lower survival rates (35). Further studies demonstrated that whilst CD8 T cell function is unchanged, CD4 T cells have an intrinsic defect in thymic selection due to blockade of epithelial CD83 interactions with thymocyte ligands (36).

Thus, CD83 expression is critical for CD4 T-cell development and has a significantly more complicated role than previously appreciated in CD4^+^ T cell peripheral function. On one hand, it behaves as an activating molecule for CD4 activation (23); whilst on the other, CD83 is highly expressed on CD4^+^ regulatory T cells for the downregulation of immune responses (32). Artificial overexpression of CD83 on naive CD4 T cells provides these cells with regulatory function. Further investigation into whether the knockdown of CD83 expression on activated T regulatory cells changes their regulatory function is required.

### B cells

CD83 is expressed at low levels by B cells once they express a functional B cell receptor (BCR). Naive B cells rapidly upregulate CD83 expression upon activation by BCR or toll-like receptor (TLR) engagement *in vitro* and *in vivo*(2,36). Engagement of CD83 by injection of anti-CD83 monoclonal antibodies (mAbs) induces a 100-fold increase in the IgG1 response *in vivo*(26). Activated T cells induce CD83 on B cells via CD40 engagement; however, it is independent of T cell receptor (TCR)/MHC binding and thus independent of the antigen-specificity of B cells (37). CD83 regulates B-cell maturation, activation and homeostasis and has been extensively reviewed elsewhere (2).

### DCs

CD83 is best known as a DC maturation marker. In mice, bone marrow-derived DCs express CD83 transcripts and lipopolysaccharide (LPS) exposure induces CD83 expression in bone marrow-derived DCs. CD83 is expressed at low levels by a small population of freshly isolated splenic and thymic CD11c^+^CD8*α*^+^ and CD11c^+^CD8*α*^−^ conventional DCs; however, it is not expressed by freshly isolated plasmacytoid DCs. Splenic and thymic conventional DCs upregulate cell surface CD83 within hours of maturation with LPS and splenic plasmacytoid DCs upregulate CD83 following overnight maturation with CpG and recombinant IL-3 (3).

CD83^−/−^ DCs derived from bone marrow (33) induce allogenic T-cell proliferation to the same extent as their wild type counterparts. Similar results are also obtained from CD83 mutant mice (34). Although the CD83^−/−^ splenic DCs from mutant CD83-deficient mice exhibit the same capacity to induce proliferation of OT-II cells (38), they show reduced IL-2 production by OT-II cells. Moreover, downregulation of CD83 expression on human DCs through RNAi results in a less potent induction of allogeneic T-cell proliferation, reduced cytokine secretion by T cells and decreased capacity in the priming of functional tumour antigen-specific CD8^+^ T cells (39,40).

In a CD83 reporter mouse, the CD83 promoter is highly active in all DCs and B cells in lymphoid organs. Promoter activity in B cells is largely determined by the stage of development and is upregulated in the late pre-B-cell stage and is maintained to a high level in all peripheral B cells. Using these knock-in mice, it was demonstrated that CD83 in B cells and DCs is mainly intracellular; however, it is upregulated following TLR stimulation. CD83 promoter activity in T cells is dependent on activation (41).

Taken together, CD83 expression on T cells, B cells and DCs is activation-dependent. Upon stimulation, CD83 is expressed or upregulated in those cells. Targeting CD83 on T cells, B cells and DCs changes the biological functions of these cells.

Activated DCs, T cells or B cells expressing CD83 interact with cells expressing putative CD83 ligand to induce the inflammatory immune cascade. This interaction may be prevented by: i) administration of biological or chemical antagonists, including a soluble recombinant CD83 protein; ii) a therapeutic antibody that binds to the ligand binding site or proximally to hinder the interaction; or iii) a cell death-inducing monoclonal antibody that binds specifically to CD83 and utilises antibody Fc effector mechanisms to initiate target cell destruction.

## 4. Soluble form of CD83 has an immune-regulatory function

A soluble form of CD83 was reported in 2001 (27). It was identified that sCD83 is released from activated DCs and B cells *in vitro*; although the mechanism of release is yet to be elucidated. Low levels of circulating sCD83 are also present in sera from normal individuals. sCD83 levels are drastically increased in patients suffering from haematological malignancies or rheumatoid arthritis (42). Following human cytomegalovirus (HCMV) infection, sCD83 is shed from the cell surface by proteolytic cleavage and subsequently blocks T-cell stimulation (43); when DCs are infected with herpes simplex virus (HSV)-1, a significant downregulation of the cell surface expression of CD83 is observed (44). These results suggest that CD83 may be targeted by different viruses to subvert the immune attack by the host.

sCD83 has also been shown to have immune suppressive functions *in vitro* and *in vivo*(45). sCD83 inhibits DC-dependent allopeptide-specific cell proliferation *in vitro*(21) and down-regulates antitumour immune response in a murine tumour model *in vivo*(46). Another study demonstrated that the administration of sCD83 significantly delays fully mismatched alloskin graft rejection (18). sCD83 is capable of attenuating DC maturation and function and inducing donor-specific allograft tolerance to prevent cardiac allograft rejections, in the absence of toxicity (47). Furthermore, peritransplant treatment with recombinant sCD83 attenuates innate and adaptive immune responses and leads to the prevention of chronic rejection in a rat renal transplant model (48).

Multiple sclerosis (MS) is an autoimmune disease of the central nervous system. Currently, a mouse model of EAE best represents human MS. It has been demonstrated that CD4 T cells play critical roles in the pathogenesis of EAE. Passive transfer of CD4 T cells isolated from EAE induced by transfer of myelin basic protein and *in vivo* depletion of CD4 T cells inhibits the induction of EAE. Previously, it has been shown that CCR6 regulates EAE pathogenesis by controlling regulatory CD4 T-cell recruitment to target tissues (49). In an EAE mouse model, three injections of sCD83 almost completely prevented the paralysis associated with EAE. Furthermore, even when the treatment was delayed until the disease symptoms were fully established, sCD83 retained the ability to reduce the paralysis and disease symptoms (50).

## 5. Targeting CD83 using antibodies *in vivo* has therapeutic effects in different animal models

A number of studies have shown that targeting CD83 has therapeutic effects in various animal models (49,51,52).

GCA is a systemic vasculitis that affects medium- and large-sized arteries. Inflammatory lesions in GCA are composed of activated CD4^+^ T cells and macrophages. IFN-*γ* has been identified as a key cytokine and plays a critical role in regulating the effector functions of tissue-infiltrating macrophages. Activation of adventitial DCs is an early and critical event in GCA that precedes the invasion of T cells and macrophages (51). In human GCA artery-severe combined immunodeficiency (SCID) mouse cells, depletion of CD83^+^ DCs using antibodies abrogates vasculitis. Adventitial DCs from patients with a subclinical variant of GCA attract, retain and activate T cells originating from the GCA lesion, while immature DCs in healthy arteries failed to stimulate T cells (51).

CD4^+^ T cells and CD8^+^ T cells are involved in the development of GVHD. APCs from recipient and donor have been shown to be responsible for the initiation and development of GVHD (53). In a human T cell-dependent peripheral blood mononuclear cell transplanted SCID (hu-SCID) model (52), treatment with CD83 antibody prevented GVHD in a dose-dependent manner and at the same time preserved the cytotoxic T-lymphocyte responses to viruses and malignant cells, which is superior to the current therapeutic manipulations which non-specifically target T cells.

## 6. Targeting CD83 for the management of graft-versus-host disease (GVHD) and possible underlying mechanisms

### Depletion of CD83^+^ DCs and/or CD83^+^-activated CD4 T cells

Targeting CD83 using a monoclonal antibody (IgG1, clonal HB15e) (51) for GCA or a polyclonal antibody (52) for GVHD has been suggested to have the outcome of depletion of CD83-positive DCs. An *in vitro* study demonstrated that the polyclonal CD83 antibody-mediated suppression of T-cell proliferation is via the NK cell-dependent antibody-dependent cell-mediated cytotoxicity (ADCC) of CD83^+^ DCs (54). Whether the *in vivo* depletion of CD83+ DCs also depends on the same mechanism requires further investigation. CD83^+^ DCs are the mature form of DCs that stimulate T-cell proliferation, while CD83^−^ DCs are usually at an immature stage; thus, auto-reactive T cell proliferation is reduced and the absolute numbers of autoreactive T cells are diminished following the administration of antibodies against CD83 (52). A previous study presented two novel mouse anti-human CD83 mAbs; 8B4 binds to a linear epitope, whereas 1E11 recognises a conformational epitope and cross-linking of 8B4; however, 1E11 with CD83-Ig augments the fusion protein-mediated inhibition of peripheral blood mononuclear cells (PBMCs) (4). Similarly, the ratio of different subtypes of DCs within the blood and in different tissues may also change following the administration of anti-CD83, affecting the type of immune response.

CD83 inhibits the proliferation and production of IL-2 and IFN-*γ* by T cells and the inhibitory effect of CD83 is mediated by monocytes. Prostaglandin E2 (PGE2), but not IL-10 or TGF-*β*, was upregulated specifically by CD83 in monocytes (4). Since activated CD4 T cells also express CD83, the administration of anti-CD83 antibodies may also deplete the activated CD4 effector T cells. Additionally, regulatory T cells expressing CD83 may also be targeted; thus, the balance between T effector cells and regulatory T cells may shift to a more regulatory response.

### Reduction of T-cell trafficking to the target organ

In a mouse EAE model, administration of sCD83 inhibited CD45^+^ cell infiltration into the brain and spinal cord. The CD45^+^ cells located in the brain and spinal cord are similar to those in naive, untreated mice (50), suggesting that the migration pattern or migration abilities to the target organ in response to different chemokines of autoreactive T cells may be different following administration of sCD83. In a hu-SCID mouse model of GCA, activated CD83^+^ DCs in the artery of a polymyalgia rheumatica (PMR) patient attracted, retained and activated autoreactive T cells. Following depletion of CD83^+^ DCs in a GVHD model, as well as in a GCA model, the reduced severity of diseases may be attributed to reduced trafficking of autoreactive CD4^+^ T cells to the target organs.

Reductions in autoreactive T cells in the target organs may be due to reductions in the absolute numbers of autoreactive T cells and depletion of CD83^+^ DCs in the target organ; the remaining immature DCs in the target organ fail to attract autoreactive T cells that are likely to damage the target organ.

### Promotion of the generation of induced regulatory T cells

Regulatory T cells are a group of T cells with regulatory functions that control immune responses against pathogens and excessive responses to self. There are numerous cell types that have regulatory functions; however, the predominant T regulatory cells are naturally occurring CD4^+^Foxp3^+^ regulatory T cells and induced CD4^+^ T regulatory cells. Anti-CD83 antibody treatment may leave tolerogenic and nonactivated CD83^−^ tolerogenic DCs; these cells may induce regulatory T cells with potential allosuppressive benefits (55). It has been demonstrated that a group of IL-10- and IFN-*γ*-secreting CD4^+^ T cells induced following repeated stimulation have regulatory functions to avoid an excessive response (56). GVHD is a disease with profound pro-inflammatory cytokine production and constant host stimulation, and IFN-*γ* is able to protect against several manifestations of GVHD in recipients of MHC-mismatched haematopoietic cells (57). Therefore, it is likely that these cells exist in GVHD and that the depletion of CD83^+^ DCs may preserve the DCs that are responsible for the generation of these cells. Additionally, as discussed above, it has been demonstrated that CD83 expression on CD4^+^T cells affects their function; therefore, an anti-CD83 antibody or sCD83 administration may also affect the CD4^+^ T cells expressing CD83. Further investigation is warranted for the contribution of depletion of CD83^+^ CD4 T cells.

Administration of anti-CD83 antibodies or sCD83 may attenuate diseases, including GVHD. Investigating the underlying mechanisms is likely to provide improved control of diseases such as GVHD.

## References

[1] Zhou LJ, Schwarting R, Smith HM, Tedder TF (1992). A novel cell-surface molecule expressed by human interdigitating reticulum cells, Langerhans cells, and activated lymphocytes is a new member of the Ig superfamily. J Immunol.

[2] Breloer M (2008). CD83: regulator of central T cell maturation and peripheral immune response. Immunol Lett.

[3] Prazma CM, Tedder TF (2008). Dendritic cell CD83: a therapeutic target or innocent bystander?. Immunol Lett.

[4] Chen L, Zhu Y, Zhang G, Gao C, Zhong W, Zhang X (2011). CD83-stimulated monocytes suppress T-cell immune responses through production of prostaglandin E2. Proc Natl Acad Sci USA.

[5] Ferrara JL, Levine JE, Reddy P, Holler E (2009). Graft-versus-host disease. Lancet.

[6] Shlomchik WD, Couzens MS, Tang CB (1999). Prevention of graft versus host disease by inactivation of host antigen-presenting cells. Science.

[7] Matte CC, Liu J, Cormier J (2004). Donor APCs are required for maximal GVHD but not for GVL. Nat Med.

[8] Reddy P, Maeda Y, Liu C, Krijanovski OI, Korngold R, Ferrara JL (2005). A crucial role for antigen-presenting cells and alloantigen expression in graft-versus-leukemia responses. Nat Med.

[9] Merad M, Hoffmann P, Ranheim E (2004). Depletion of host Langerhans cells before transplantation of donor alloreactive T cells prevents skin graft-versus-host disease. Nat Med.

[10] Markey KA, Banovic T, Kuns RD (2009). Conventional dendritic cells are the critical donor APC presenting alloantigen after experimental bone marrow transplantation. Blood.

[11] Strober S, Lowsky R (2012). Rare cells predict GVHD. Blood.

[12] Gowdy KM, Cardona DM, Nugent JL (2012). Novel role for surfactant protein A in gastrointestinal graft-versus-host disease. J Immunol.

[13] Banovic T, Markey KA, Kuns RD (2009). Graft-versus-host disease prevents the maturation of plasmacytoid dendritic cells. J Immunol.

[14] Horváth R, Budinský V, Kayserová J (2009). Kinetics of dendritic cells reconstitution and costimulatory molecules expression after myeloablative allogeneic haematopoetic stem cell transplantation: implications for the development of acute graft-versus host disease. Clin Immunol.

[15] Reshef R, Luger SM, Hexner EO (2012). Blockade of lymphocyte chemotaxis in visceral graft-versus-host disease. N Engl J Med.

[16] Levine JE, Logan BR, Wu J (2012). Acute graft-versus-host disease biomarkers measured during therapy can predict treatment outcomes: a Blood and Marrow Transplant Clinical Trials Network study. Blood.

[17] MacMillan ML, DeFor TE, Weisdorf DJ (2012). What predicts high risk acute graft-versus-host disease (GVHD) at onset?: identification of those at highest risk by a novel acute GVHD risk score. Br J Haematol.

[18] Xu J, Racke MK, Drew PD (2007). Peroxisome proliferator-activated receptor-alpha agonist fenofibrate regulates IL-12 family cytokine expression in the CNS: relevance to multiple sclerosis. J Neurochem.

[19] Lechmann M, Kremmer E, Sticht H, Steinkasserer A (2002). Overexpression, purification, and biochemical characterization of the extracellular human CD83 domain and generation of monoclonal antibodies. Protein Expr Purif.

[20] Cao W, Lee SH, Lu J (2005). CD83 is preformed inside monocytes, macrophages and dendritic cells, but it is only stably expressed on activated dendritic cells. Biochem J.

[21] Lechmann M, Krooshoop DJ, Dudziak D (2001). The extracellular domain of CD83 inhibits dendritic cell-mediated T cell stimulation and binds to a ligand on dendritic cells. J Exp Med.

[22] Davis SJ, Davies EA, Barclay AN (1995). Ligand binding by the immunoglobulin superfamily recognition molecule CD2 is glycosylation-independent. J Biol Chem.

[23] Su LL, Iwai H, Lin JT, Fathman CG (2009). The transmembrane E3 ligase GRAIL ubiquitinates and degrades CD83 on CD4 T cells. J Immunol.

[24] Piper RC, Luzio JP (2007). Ubiquitin-dependent sorting of integral membrane proteins for degradation in lysosomes. Curr Opin Cell Biol.

[25] Hegde AN, DiAntonio A (2002). Ubiquitin and the synapse. Nat Rev Neurosci.

[26] Kretschmer B, Lüthje K, Schneider S, Fleischer B, Breloer M (2009). Engagement of CD83 on B cells modulates B cell function in vivo. J Immunol.

[27] Hock BD, Kato M, McKenzie JL, Hart DN (2001). A soluble form of CD83 is released from activated dendritic cells and B lymphocytes, and is detectable in normal human sera. Int Immunol.

[28] Dudziak D, Nimmerjahn F, Bornkamm GW, Laux G (2005). Alternative splicing generates putative soluble CD83 proteins that inhibit T cell proliferation. J Immunol.

[29] Colonna M, Nakajima H, Navarro F, López-Botet M (1999). A novel family of Ig-like receptors for HLA class I molecules that modulate function of lymphoid and myeloid cells. J Leukoc Biol.

[30] Arulanandam AR, Withka JM, Wyss DF (1993). The CD58 (LFA-3) binding site is a localized and highly charged surface area on the AGFCC’C” face of the human CD2 adhesion domain. Proc Natl Acad Sci USA.

[31] Nakaishi A, Hirose M, Yoshimura M (2008). Structural insight into the specific interaction between murine SHPS-1/SIRP alpha and its ligand CD47. J Mol Biol.

[32] Reinwald S, Wiethe C, Westendorf AM (2008). CD83 expression in CD4+ T cells modulates inflammation and autoimmunity. J Immunol.

[33] Fujimoto Y, Tu L, Miller AS (2002). CD83 expression influences CD4+ T cell development in the thymus. Cell.

[34] Garcia-Martinez LF, Appleby MW, Staehling-Hampton K (2004). A novel mutation in CD83 results in the development of a unique population of CD4^+^ T cells. J Immunol.

[35] Lüthje K, Cramer SO, Ehrlich S (2006). Transgenic expression of a CD83-immunoglobulin fusion protein impairs the development of immune-competent CD4-positive T cells. Eur J Immunol.

[36] Prazma CM, Yazawa N, Fujimoto Y, Fujimoto M, Tedder TF (2007). CD83 expression is a sensitive marker of activation required for B cell and CD4^+^ T cell longevity in vivo. J Immunol.

[37] Kretschmer B, Kuhl S, Fleischer B, Breloer M (2011). Activated T cells induce rapid CD83 expression on B cells by engagement of CD40. Immunol Lett.

[38] Kretschmer B, Lüthje K, Ehrlich S (2008). CD83 on murine APC does not function as a costimulatory receptor for T cells. Immunol Lett.

[39] Prechtel AT, Turza NM, Theodoridis AA, Steinkasserer A (2007). CD83 knockdown in monocyte-derived dendritic cells by small interfering RNA leads to a diminished T cell stimulation. J Immunol.

[40] Aerts-Toegaert C, Heirman C, Tuyaerts S (2007). CD83 expression on dendritic cells and T cells: correlation with effective immune responses. Eur J Immunol.

[41] Lechmann M, Shuman N, Wakeham A, Mak TW (2008). The CD83 reporter mouse elucidates the activity of the CD83 promoter in B, T, and dendritic cell populations in vivo. Proc Natl Acad Sci USA.

[42] Hock BD, O’Donnell JL, Taylor K (2006). Levels of the soluble forms of CD80, CD86, and CD83 are elevated in the synovial fluid of rheumatoid arthritis patients. Tissue Antigens.

[43] Sénéchal B, Boruchov AM, Reagan JL, Hart DN, Young JW (2004). Infection of mature monocyte-derived dendritic cells with human cytomegalovirus inhibits stimulation of T-cell proliferation via the release of soluble CD83. Blood.

[44] Kruse M, Rosorius O, Krätzer F (2000). Inhibition of CD83 cell surface expression during dendritic cell maturation by interference with nuclear export of CD83 mRNA. J Exp Med.

[45] Zinser E, Steinkasserer A (2008). Published studies reporting the efficacy of soluble CD83 in vitro as well as in vivo. Immunol Lett.

[46] Scholler N, Hayden-Ledbetter M, Dahlin A, Hellstrom I, Hellstrom KE, Ledbetter JA (2002). Cutting edge: CD83 regulates the development of cellular immunity. J Immunol.

[47] Ge W, Arp J, Lian D (2010). Immunosuppression involving soluble CD83 induces tolerogenic dendritic cells that prevent cardiac allograft rejection. Transplantation.

[48] Lan Z, Ge W, Arp J (2010). Induction of kidney allograft tolerance by soluble CD83 associated with prevalence of tolerogenic dendritic cells and indoleamine 2,3-dioxygenase. Transplantation.

[49] Villares R, Cadenas V, Lozano M (2009). CCR6 regulates EAE pathogenesis by controlling regulatory CD4^+^ T-cell recruitment to target tissues. Eur J Immunol.

[50] Zinser E, Lechmann M, Golka A, Lutz MB, Steinkasserer A (2004). Prevention and treatment of experimental autoimmune encephalomyelitis by soluble CD83. J Exp Med.

[51] Ma-Krupa W, Jeon MS, Spoerl S, Tedder TF, Goronzy JJ, Weyand CM (2004). Activation of arterial wall dendritic cells and breakdown of self-tolerance in giant cell arteritis. J Exp Med.

[52] Wilson J, Cullup H, Lourie R (2009). Antibody to the dendritic cell surface activation antigen CD83 prevents acute graft-versus-host disease. J Exp Med.

[53] Holler E, Rogler G, Brenmoehl J (2006). Prognostic significance of NOD2/CARD15 variants in HLA-identical sibling hematopoietic stem cell transplantation: effect on long-term outcome is confirmed in 2 independent cohorts and may be modulated by the type of gastrointestinal decontamination. Blood.

[54] Munster DJ, MacDonald KP, Kato M, Hart DJ (2004). Human T lymphoblasts and activated dendritic cells in the allogeneic mixed leukocyte reaction are susceptible to NK cell-mediated anti-CD83-dependent cytotoxicity. Int Immunol.

[55] Jonuleit H, Tüting T, Steitz J (2000). Efficient transduction of mature CD83^+^ dendritic cells using recombinant adenovirus suppressed T cell stimulatory capacity. Gene Ther.

[56] Chen J, Liu XS (2009). Development and function of IL-10 IFN-gamma-secreting CD4(+) T cells. J Leukoc Biol.

[57] Delisle JS, Gaboury L, Bélanger MP, Tassé E, Yagita H, Perreault C (2008). Graft-versus-host disease causes failure of donor hematopoiesis and lymphopoiesis in interferon-gamma receptor-deficient hosts. Blood.

